# The Value of Cytology Smears for* Acanthamoeba* Keratitis

**DOI:** 10.1155/2016/4148968

**Published:** 2016-06-15

**Authors:** Sangita P. Patel, Jamie L. Schaefer, Ryan Jaber, Joyce Paterson, Weiguo Liu, Federico Gonzalez-Fernandez

**Affiliations:** ^1^Department of Ophthalmology, Ross Eye Institute, The State University of New York at Buffalo, Jacobs School of Medicine and Biomedical Sciences, 1176 Main Street, Buffalo, NY 14209, USA; ^2^SUNY Eye Institute, Buffalo, NY 14214, USA; ^3^Research Service, Veterans Administration Western New York Healthcare System (VAWNYHS), Building 20, 3495 Bailey Avenue, Buffalo, NY 14215, USA; ^4^Department of Pathology, Buffalo General Medical Center, Kaleida Health, 100 High Street, Buffalo, NY 14203, USA; ^5^Department of Pathology, The State University of New York at Buffalo, Jacobs School of Medicine and Biomedical Sciences, 206 Farber Hall, Buffalo, NY 14214, USA; ^6^Departments of Ophthalmology and Pathology, University of Mississippi Medical Center, 2500 North State Street, Jackson, MS 39216, USA; ^7^Research & Development Service, G.V. (Sonny) Montgomery Veterans Affair Medical Center, 1500 East Woodrow Wilson Avenue, Jackson, MS 39216, USA

## Abstract

*Purpose*.* Acanthamoeba* keratitis remains a difficult diagnosis despite advances in genetic and imaging technologies. The purpose of this paper is to highlight the utility of cytology smears for diagnosis of* Acanthamoeba* keratitis.* Methods*. This is a case study of the diagnostic course for a patient with suspected* Acanthamoeba* keratitis.* Results*. A 40-year-old male with poor contact lens hygiene presented with severe left eye pain. Slit lamp examination showed two peripheral ring infiltrates without an epithelial defect. The epithelium over both infiltrates was removed with a Kimura spatula. Half of the sample was smeared on a dry microscope slide and the other half was submitted for* Acanthamoeba* culture and PCR. Both culture and PCR were negative for* Acanthamoeba*, but hematoxylin and eosin stain of the smear revealed double-walled cysts.* Conclusion*. H&E staining of corneal cytology specimens is an efficient and readily available test for diagnosis of* Acanthamoeba* keratitis.

## 1. Introduction

*Acanthamoeba* keratitis is an uncommon cause of infectious keratitis that often goes undiagnosed until later stages of disease. The delay in diagnosis and appropriate treatment allows progression of disease with consequent visual morbidity. However, even when clinical findings suggest the diagnosis, confirmatory testing is challenging. Cultures for* Acanthamoeba* grow slowly and are often negative [1]. Although polymerase chain reaction (PCR) analysis of ocular specimens for* Acanthamoeba* has become more standardized, recent reports demonstrate limited utility compared to culture [2].* In vivo* confocal microscopy of the cornea is useful for rapid and noninvasive identification of the double-walled cysts characteristic of* Acanthamoeba*, but instrumentation is expensive and not readily available [3]. We present a case to highlight the power of inexpensive, readily available cytology preparations for the rapid diagnosis of* Acanthamoeba* keratitis.

## 2. Case Presentation

**Figure 1:**
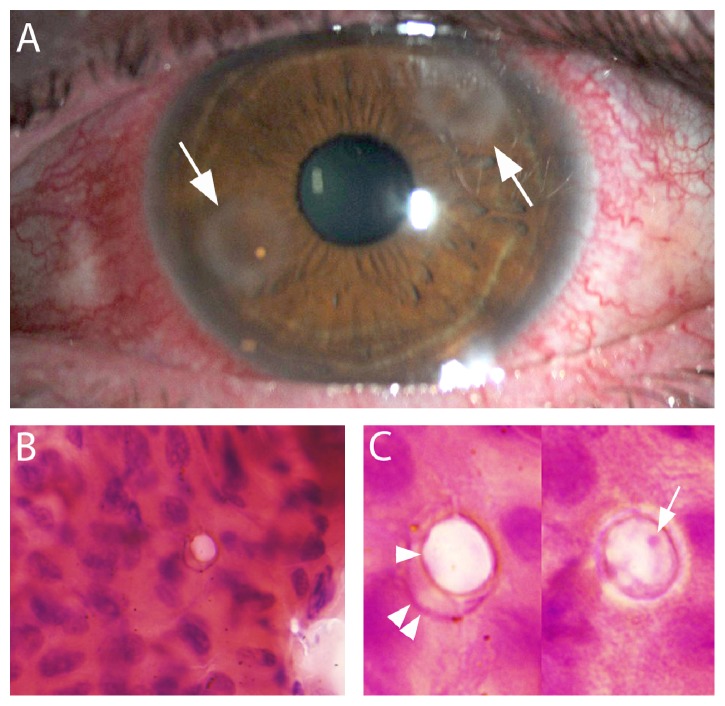
*Acanthamoeba* keratitis. (A) Photograph depicting the two peripheral corneal-ring infiltrates (arrows) in this patient. (B) Low magnification photomicrograph of H&E stained corneal epithelium scraped from the areas of infiltrates shown in panel (A). A double-walled cyst characteristic of* Acanthamoeba* is appreciated against the cellular background. (C) Higher magnification views of the cyst at two focal planes demonstrate the presence of the* Acanthamoeba* exocyst (double arrow head), endocyst (single arrowhead), and nucleolus (arrow).

A 40-year-old man with history of poor contact lens hygiene had onset of left eye redness, photophobia, irritation, and 8/10 pain. After a week of unsuccessful treatment with an unspecified antibiotic eye drop, his treatment was switched to moxifloxacin 4x/day and prednisolone acetate 1% 4x/day. He was referred to our clinic two weeks following the onset of symptoms. His presenting visual acuity was 20/20 in the right eye and 20/25 in the left eye. On examination, two peripheral ring infiltrates were present on his left cornea without any epithelial irregularity. Prednisolone eye drops were discontinued given the high clinical suspicion for* Acanthamoeba* keratitis. The following week, examination showed granularity of the epithelium overlying the ring infiltrates with an increase in stromal inflammation (Figure 1(A)). The corneal epithelium over the infiltrates was removed with a Kimura spatula. Half of the sample was submitted for* Acanthamoeba* culture and PCR and the other half smeared directly on a dry microscope slide for routine cytology. The smear was allowed to air-dry. No fixative step was used.

Both* Acanthamoeba* culture and PCR were negative; however, hematoxylin and eosin (H&E) staining of the epithelial smear demonstrated the presence of double-walled cysts typical of* Acanthamoeba* keratitis (Figure 1, (B) and (C)). Another corneal epithelial debridement was performed and submitted for culture and cytology after 10 weeks of therapy with Chlorhexidine 0.02%, Neosporin, and Cyclopentolate 1% ophthalmic solutions. Culture remained negative; however, the cytology specimen again revealed the presence of double-walled cysts. Occasional single walled structures, representing single walled exocysts, were also seen. Electron microscopy studies indicate that exocystation is accompanied by at least partial enzymatic digestion of the endocyst [4]. This would explain the single wall appearance of the empty cysts. Also, such exocysts would not be expected to support growth in microbiological culture (as noted in this case) or contain genomic DNA for PCR detection.

Following 4 months of therapy with Chlorhexidine 0.02% and Neosporin, the patient's eye pain decreased and the cornea showed stable stromal scarring. Therapy was gradually tapered without recurrence.

## 3. Discussion

In this case, high clinical suspicion and positive cytology allowed rapid diagnosis and initiation of appropriate therapy for* Acanthamoeba* keratitis. High clinical suspicion is essential for the diagnosis of* Acanthamoeba* keratitis with a positive predictive value of 89% [5]. However, confirmatory testing is necessary. Cytology services are present in most clinical centers and are therefore more accessible than facilities for* in vivo* corneal confocal microscopy,* Acanthamoeba* culture, or PCR. Cytological identification of the cyst does not require living organisms as needed for culture or intact DNA as required for PCR [6]. The value of cytology was well documented two decades ago during an outbreak of* Acanthamoeba* keratitis [7]. In that study, culture was negative in all of the specimens evaluated, but cytology was positive in over 80% of suspected cases. Almost half of cases equivocal for* Acanthamoeba* by* in vivo* confocal microscopy were positive on H&E stained cytology specimens. Other methods of staining that have been described for detection of* Acanthamoeba* include lactophenol-cotton blue, acridine orange, calcofluor white, and silver stain [8]. However, we decided to stain the smear with H&E as recent literature suggests that H&E is more sensitive and specific than other stains, particularly calcofluor white [9]. The cytological identification of* Acanthamoeba* has been underutilized in recent years perhaps due to the introduction of new molecular and imaging methods. In fact, there are only a limited number of descriptions of the cytological features of these organisms in smears [6, 10]. A summary of the pros and cons of diagnostic tests for* Acanthamoeba* keratitis is presented in Table 1.

*Acanthamoeba* keratitis requires a multifaceted diagnostic approach. The value of cytology for efficient diagnosis should not be underestimated. Close collaboration between the ophthalmologist and pathologist is key to obtaining an informative cytological study.

## Figures and Tables

**Table 1:** Comparison of diagnostic approaches for *Acanthamoeba* keratitis.

Diagnostic modality	Advantages	Disadvantages
Microbiological culture	(i) Direct identification	(i) Low sensitivity [1] (ii) Can take up to 1 week

Polymerase chain reaction (PCR)	(i) Specific (ii) Fast	(i) Requires intact DNA [2] (ii) Not readily available

*In vivo* confocal microscopy	(i) Immediate identification of double-walled cysts (ii) Noninvasive	(i) Not readily available (ii) Requires trained observer to recognize cysts in images [1]

Histopathology	(i) Specific (ii) Requires several days for diagnosis (iii) Multiple stains and/or immunoperoxidase studies can be done	(i) Requires significant tissue (corneal biopsy or keratoplasty specimen)

Cytological smear	(i) Minimally invasive (ii) Identifies both empty and double-walled cyst (iii) Fast (iv) Biopsy easy to perform	(i) Organisms in deep stroma not easily represented

Electron microscopy	(i) Specific	(i) Requires weeks to process (ii) Expensive and labor intensive (iii) Practical only for small tissue samples
